# Highly Improved Solar Energy Harvesting for Fuel Production from CO_2_ by a Newly Designed Graphene Film Photocatalyst

**DOI:** 10.1038/s41598-018-35135-7

**Published:** 2018-11-13

**Authors:** Rajesh K. Yadav, Jeong-O Lee, Abhishek Kumar, No-Joong Park, Dolly Yadav, Jae Young Kim, Jin-Ook Baeg

**Affiliations:** Artificial Photosynthesis Research Group, Korea Research Institute of Chemical Technology (KRICT), 100 Jang-dong, Yuseong, Daejeon 305 600 Republic of Korea

## Abstract

Our growing energy demands must be met by a sustainable supply with reduced carbon intensity. One of the most exciting prospects to realize this goal is the photocatalyst-biocatalyst integrated artificial photosynthesis system which affords solar fuel/chemicals in high selectivity from CO_2_. Graphene based photocatalysts are highly suitable for the system, but their industrial scale use requires immobilization for improved separation and recovery of the photocatalyst. Therefore for practical purposes, design and fabrication of film type graphene photocatalyst with higher solar energy conversion efficiency is an absolute necessity. As a means to achieve this, we report herein the successful development of a new type of flexible graphene film photocatalyst that leads to >225% rise in visible light harvesting efficiency of the resultant photocatalyst-biocatalyst integrated artificial photosynthesis system for highly selective solar fuel production from CO_2_ compared to conventional spin coated graphene film photocatalyst. It is an important step towards the design of a new pool of graphene film based photocatalysts for artificial photosynthesis of solar fuels from CO_2_.

## Introduction

Graphene is as an ideal material for light harvesting applications^1–9^ owing to its exceptional physical properties, including ultrahigh electron mobility^8^, and ballistic transport^10^. This has triggered keen interest in the development of graphene based nanoparticle powder or nanocomposite photocatalysts^1–7^. However the industrial scale application of these photocatalysts is largely limited due to difficulty in their filtration and separation. Therefore for practical and economic purposes design and fabrication of film type graphene photocatalyst with higher solar energy conversion efficiency is an absolute necessity.

Graphene films have been previously fabricated by spin coating method^11–15^. Although easy to formulate, uncontrolled capillary flow and dewetting processes during solvent evaporation result in non-uniform films with random orientation of graphene-chromophore units leading to increased charge recombination and consequently lower photocatalytic activity. Moreover, chromophore coupling to graphene sheet is usually carried out through functional groups such as –COOH present on graphene oxide (GO) or its reduced forms such as chemically converted graphene (CCG)^3,16^. They contain a large number of non-conjugated tetrahedral sp^3^ carbons which lie slightly above or below the graphene plane, further limiting electronic conductivity of the photocatalyst^14,17^. Keeping in view these crucial bottlenecks, it is imperative to develop a new design and fabrication strategy for graphene film photocatalysts.

**Figure 1:**
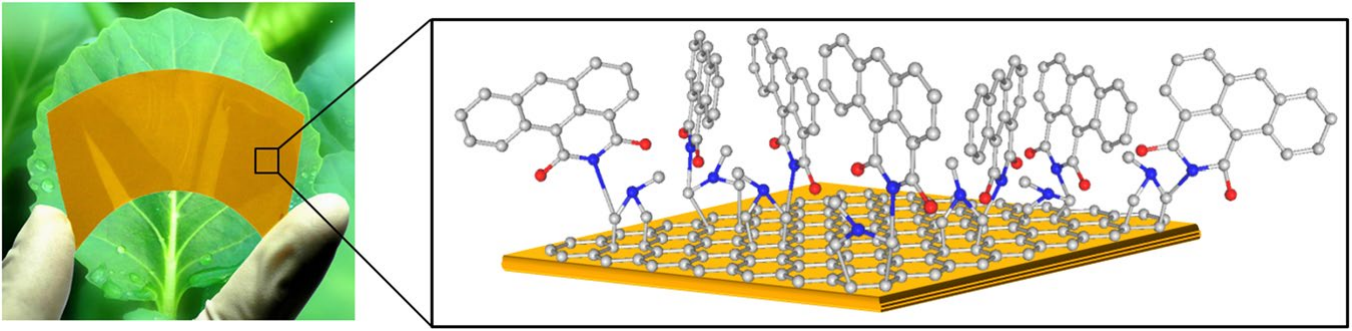
Photograph of GFPC 1 along with the detailed structure (gray = C; red = O; blue = N).

**Figure 2:**
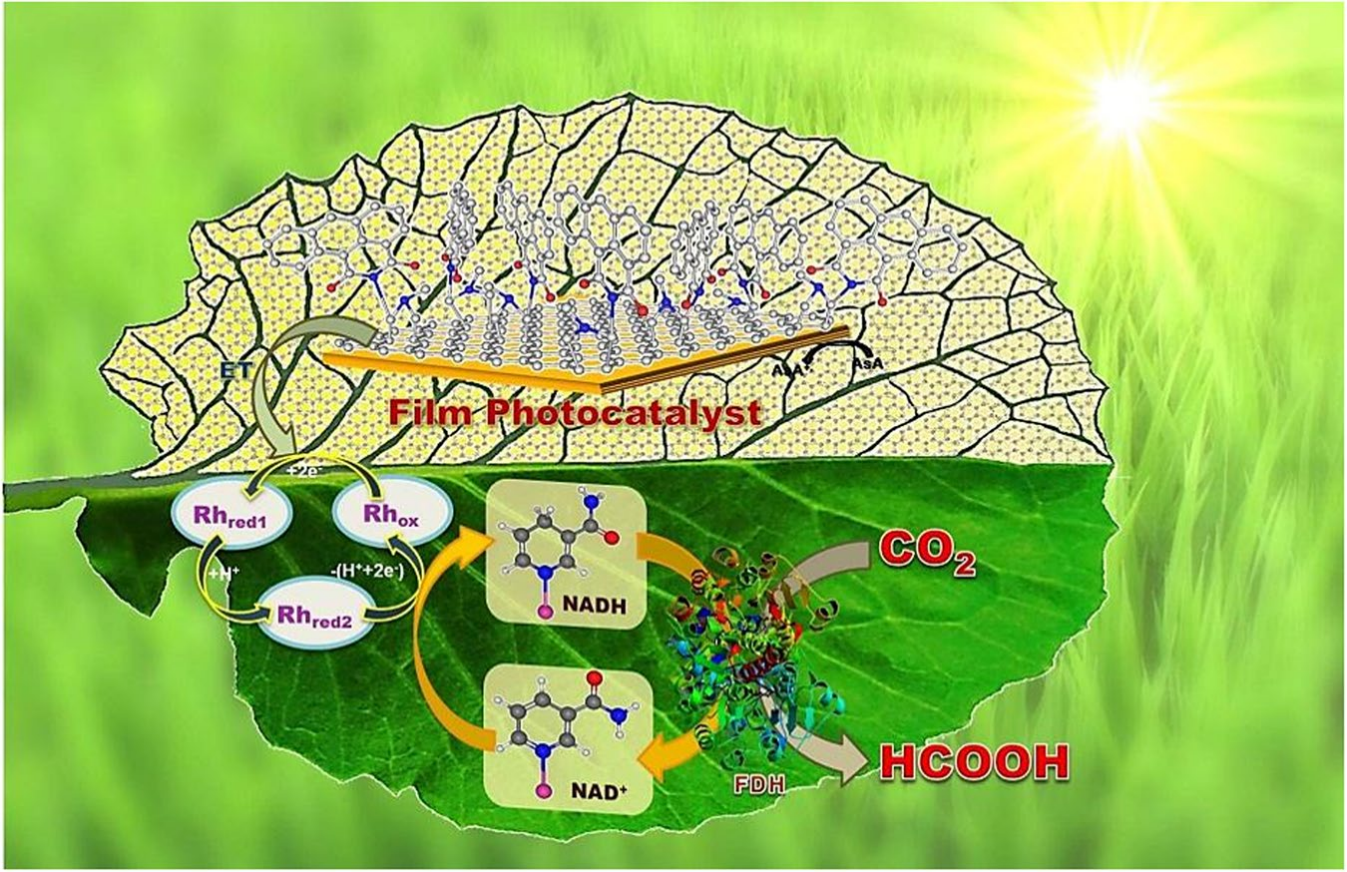
Schematic illustration of highly selective formic acid production from CO_2_ by the graphene film based photocatalyst-biocatalyst integrated artificial photosynthesis system. FDH = Formate Dehydrogenase, Rh_ox_ = [Cp*Rh(bipy)H_2_O]^2+^, Rh_red1_ = Cp*Rh(bipy), Rh_red2_ = [Cp*Rh(bipy)H]^+^; Cp* = pentamethylcyclopentadienyl, bpy = 2,2ʹ-bipyridine.

Here we report the successful development of a new type of flexible graphene film photocatalyst (hereinafter GFPC 1; Fig. 1) that leads to a 2.3 fold rise in visible light harvesting efficiency of the resultant photocatalyst-biocatalyst integrated artificial photosynthesis system (Fig. 2) for highly selective solar fuel production from CO_2_ compared to conventional spin coated graphene film photocatalyst. The monolayer graphene for this work was supported on a flexible polyimide sheet leading to a practical and easy to use photocatalyst. It also helped to solve the problem of insufficient mechanical strength of monolayer graphene film photocatalyst. The lack of functional groups on graphene monolayer for chromophore attachment presented another major challenge. This was ingeniously overcome by attaching chromophore units to sp^2^ carbon centers on graphene *via* 1,3-dipolar cycloaddition reaction. To the best of our knowledge, graphene film photocatalyst for solar fuel production using this approach has not been fabricated prior to this work.

Our design strategy elaborated above ensured a regular arrangement of light harvesting units with spatial orientation towards visible light for improved light harvesting capability. At the same time it also ensured improved π electron delocalization, reduced recombination probability of photoexcited charge carriers and direct electron transfer from covalently attached chromophore to graphene. As a consequence the graphene film based photocatalyst exhibited higher photocatalytic activity for solar fuel production from CO_2_ in comparison to conventional spin coated sample.

One may assume that this work is conceptually similar to the literature examples. However one must note that the current protocol is more environmentally friendly as it eliminates the use of thionyl chloride which was essential for preparing the previously reported graphene-chromophore photocatalyst. It is equally important to note that here that the graphene and chromophore were bonded together previously by amide bond. On the other hand, in the current protocol direct covalent attachment of the chromophore to the sp^2^ carbon centers on graphene occurred. These advantages make the newly reported graphene film based photocatalyst highly practical with vastly improved visible light harvesting efficiency than the previously reported graphene-chromophore systems.

## Results and Discussion

### Photocatalyst-biocatalyst integrated artificial photosynthesis system for formic acid production from CO_2_

Figure 2 depicts the photocatalyst-biocatalyst integrated artificial photosynthesis system for formic acid production from CO_2_. The light-harvesting DdIC chromophore (as an electron donor) collects incident photons as an electronic transition between localized orbital around it (HOMO to LUMO) and conducts *via* graphene (as a multi electron acceptor) to reduce the rhodium complex **Rh** ([Cp*Rh(bpy)H_2_O]^2+^;Cp* = pentamethylcyclopentadienyl, bpy = 2,2′-bipyridine). Upon reduction, the rhodium complex abstracts a proton from aqueous solution and transfers hydride ion to NAD^+^, which gets converted into NADH, thus completing the photocatalytic cycle. In this way, **Rh** behaves as an electron mediator between the graphene film photocatalyst and NAD^+^ leading to regeneration of NADH cofactor. Finally the enzymatic (Formate Dehydrogenase) conversion of CO_2_ substrate to formic acid consumes NADH. The NAD^+^ thus released again acts as a substrate for photocatalytic cycle, leading to photoregeneration of NADH. The photocatalytic-enzymatic cycles thus couple integrally, leading to exclusive formic acid production from CO_2_^3^.

### Preparation and characterization of GFPC 1 photocatalyst

The GFPC 1 photocatalyst for this research work was obtained by coupling graphene film (hereinafter GF) with 1,3-Dioxo-1H-dibenzo[de,h]isoquinoline-2[3 H]-carbaldehyde (hereinafter DdIC chromophore) *via* 1,3-dipolar cycloaddition. More details are provided in the experimental section. The integration of GFPC 1 photocatalyst with biocatalyst (formate dehydrogenase enzyme) afforded the integrated artificial photosynthesis system for carrying out highly selective formic acid production from CO_2_ (Fig. 2).

**Figure 3:**
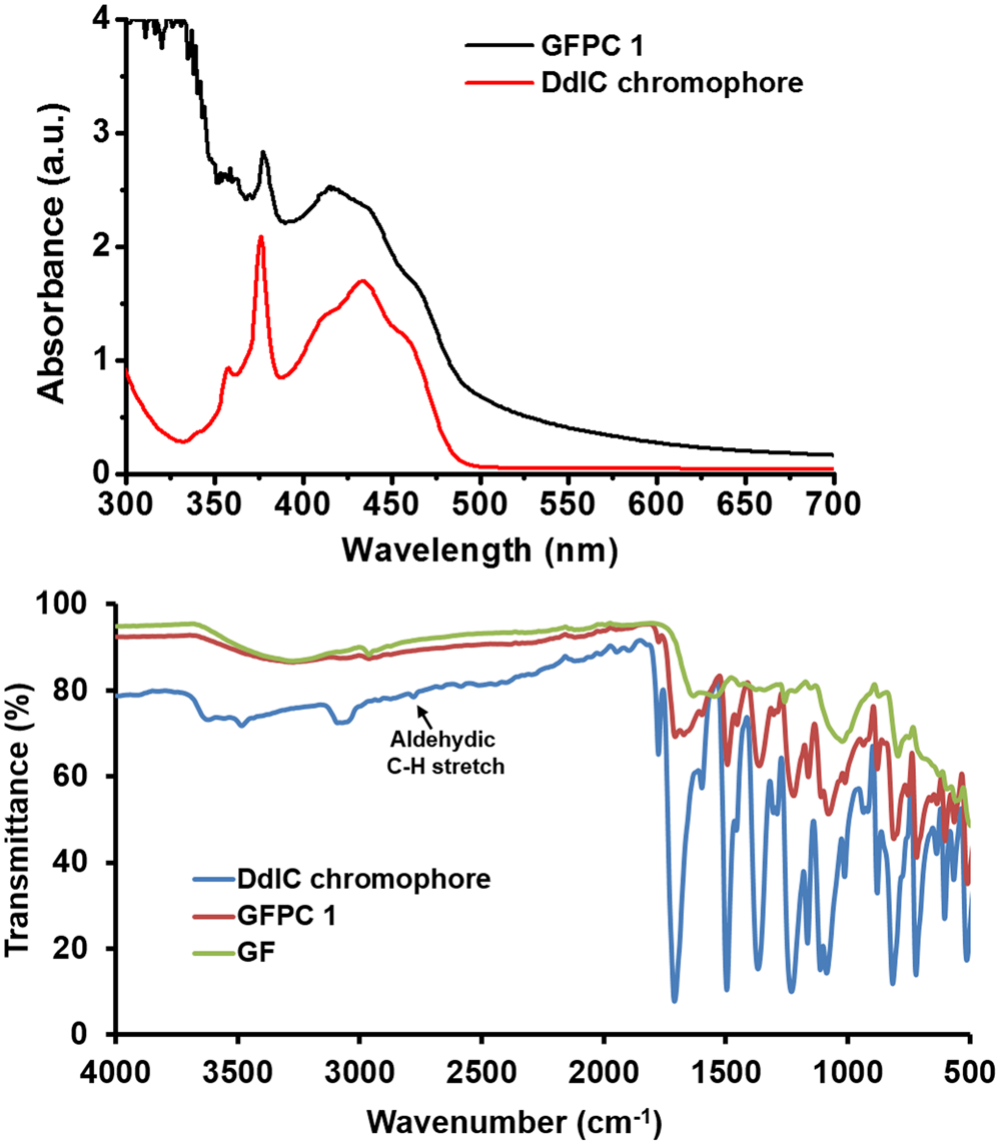
(**a**) Absorption spectrum of GFPC 1 and DdIC chromophore. (**b**) FTIR spectra of DdIC chromophore, GFPC 1 and GF.

The existence of DdIC in GFPC 1 was confirmed by UV-vis spectroscopy. A strong soret band at 433 nm was observed in the absorption spectrum of DdIC chromophore in DMF (Fig. 3a). On the other hand, the absorption spectrum of GFPC 1 exhibited a broad peak from 400–470 nm with a blue shifted maxima of DdIC unit (415 nm)^18^. This blue shift in absorption maxima can be attributed to the 1,3-dipolar cycloaddition of DdIC to GF. This observation indirectly suggested the covalent attachment of DdIC chromophores on the monolayer graphene.

The Fourier Transform Infrared (FTIR) data provided clear evidence for the coupling of chromophore to graphene film (Fig. 3b). The GF spectrum was nearly featureless, while in GFPC 1 some features of DdIC chromophore were observed in the fingerprint region (1500–550 cm^−1^)^19^. Moreover in GFPC 1, the C=O stretching was observed at 1772 and 1707 cm^−1^ while C=C stretching was observed at 1670 and 1597 cm^−1^ (Fig. S1). In comparison the C=O stretching was observed at 1775 and 1710 cm^−1^ while C=C stretching was observed at 1597 cm^−1^ in case of DdIC chromophore. The coupling was further confirmed from the C-H stretching vibration of aldehyde group at 2783 cm^−1^ in DdIC. The absence of this peak in GFPC 1 provided decisive evidence for the coupling of DdIC chromophore with GF.

**Figure 4:**
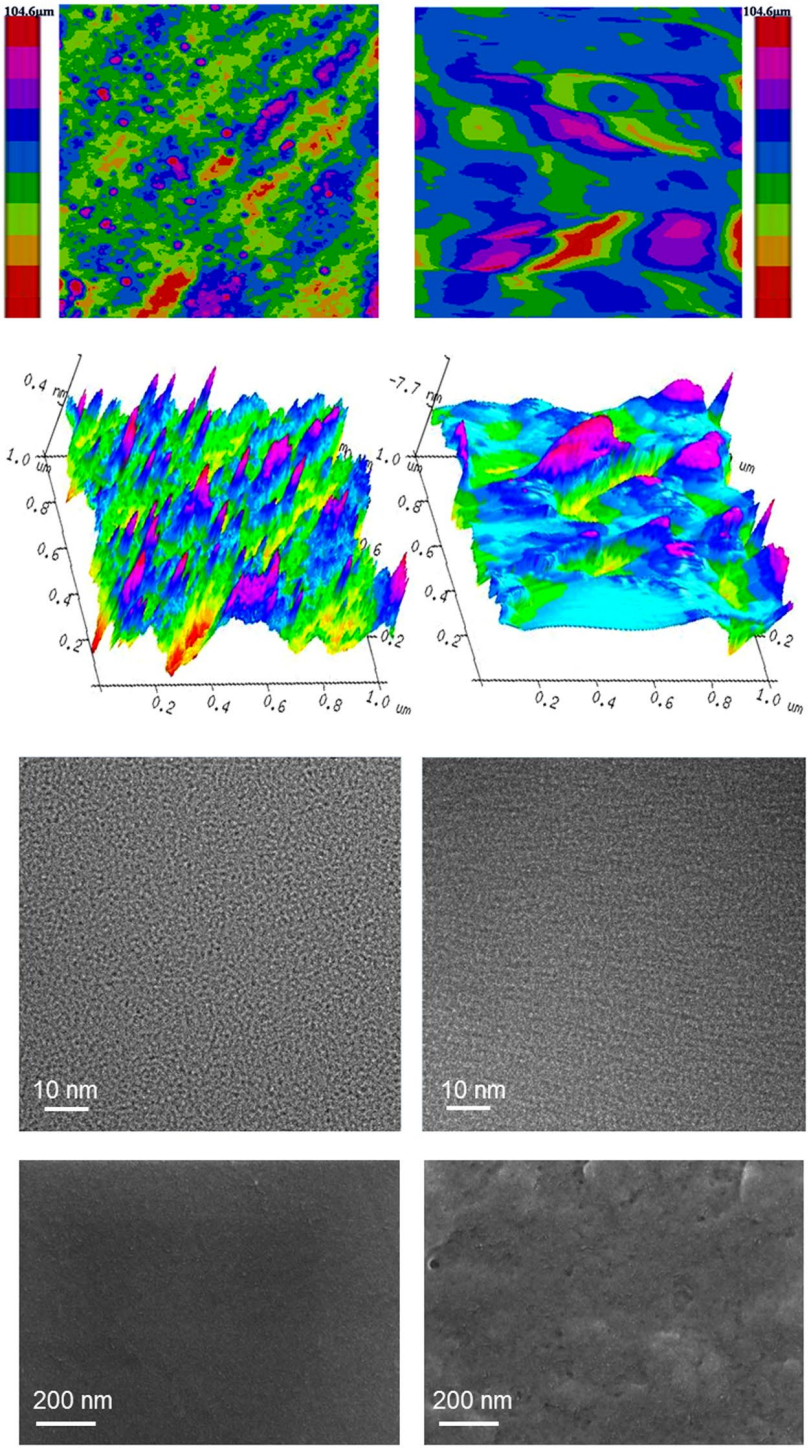
(**a**) AFM roughness image of GF. (**b**) AFM roughness image of GFPC 1. (**c**) AFM 3D image of GF. (**d**) AFM 3D image of GFPC 1. (**e**) HRTEM image of GF. (**f**) HRTEM image of GFPC 1. (**g**) SEM image of GF. (**h**) SEM image of GFPC 1.

The AFM analysis (Fig. 4) of GF revealed monolayer graphene with thickness of 0.9344 nm with identical values reported in literature (0.6–0.9 nm)^19^. An increased thickness of 1.4681 nm in GFPC 1 indicated functionalization on the GF surface. Apart from increased thickness, the presence of DdIC on the GF surface was further supported by the bloated appearance and roughness of the sample. The difference in the high resolution transmission electron microscopy (HRTEM; Fig. 4) and scanning electron microscopy (SEM; Fig. 4) images of GF and GFPC 1 further support the AFM data. Clearly evident morphological changes between GF and GFPC 1 were observed which can be attributed to the 1,3-dipolar cycloaddition of DdIC chromophore on GF surface affording the GFPC 1 photocatalyst.

Raman studies further confirmed the coupling of DdIC chromophore to GF (Fig. S3). The G and 2D bands of GF were observed at 1580 and 2670 cm^−1^, respectively^20^. Upon coupling of DdIC chromophore to GF, the G and 2D bands in GFPC 1 shifted to 1569 and 2686 cm^−1^, respectively. This shift in G and 2D bands clearly result from functionalization of GF by 1,3-dipolar cycloaddition of DdIC chromophores^21,22^. Besides this the appearance of peak at 1341 cm^−1^ in case of GFPC 1 due to D band of graphene can be further attributed to covalent bond formation between graphene film (GF) and DdIC chromophore^3,23^.

While there was no peak in N1s XPS spectrum of GF, following 1,3-dipolar cycloaddition coupling^24^ of DdIC a single new peak at 397 eV ascribed to N atoms of the C-N bond appeared (Fig. S4)^25^. Moreover changes were also observed in C1s XPS spectra of GFPC 1 when compared to GF (Fig. S5). These XPS data along with the various spectroscopic and structural data confirmed the grafting of DdIC on GF thereby resulting in GFPC 1.

Following the above analysis, the thermal behavior of CVD grown graphene film (GF) and GFPC 1 was also studied by thermogravimetric analysis (TGA) (Fig. S6). TGA was performed at a heating rate of 10 °C min^−1^ under nitrogen in the temperature range of 50–900 °C. The TGA curve for GF exhibited slow weight loss with an increase in temperature up to 900 °C. On the other hand, 24.63% weight loss due to the DdIC chromophore attached to GF was observed in GFPC 1 from 200 to 600 °C. Accordingly, the loading of DdIC chromophore in GFPC 1 was also determined by TGA and estimated to be one DdIC group per 70 carbon atoms of graphene in GFPC 1^21,26^.

### Photocatalytic NADH Regeneration and Formic Acid Production from CO_2_

**Figure 5:**
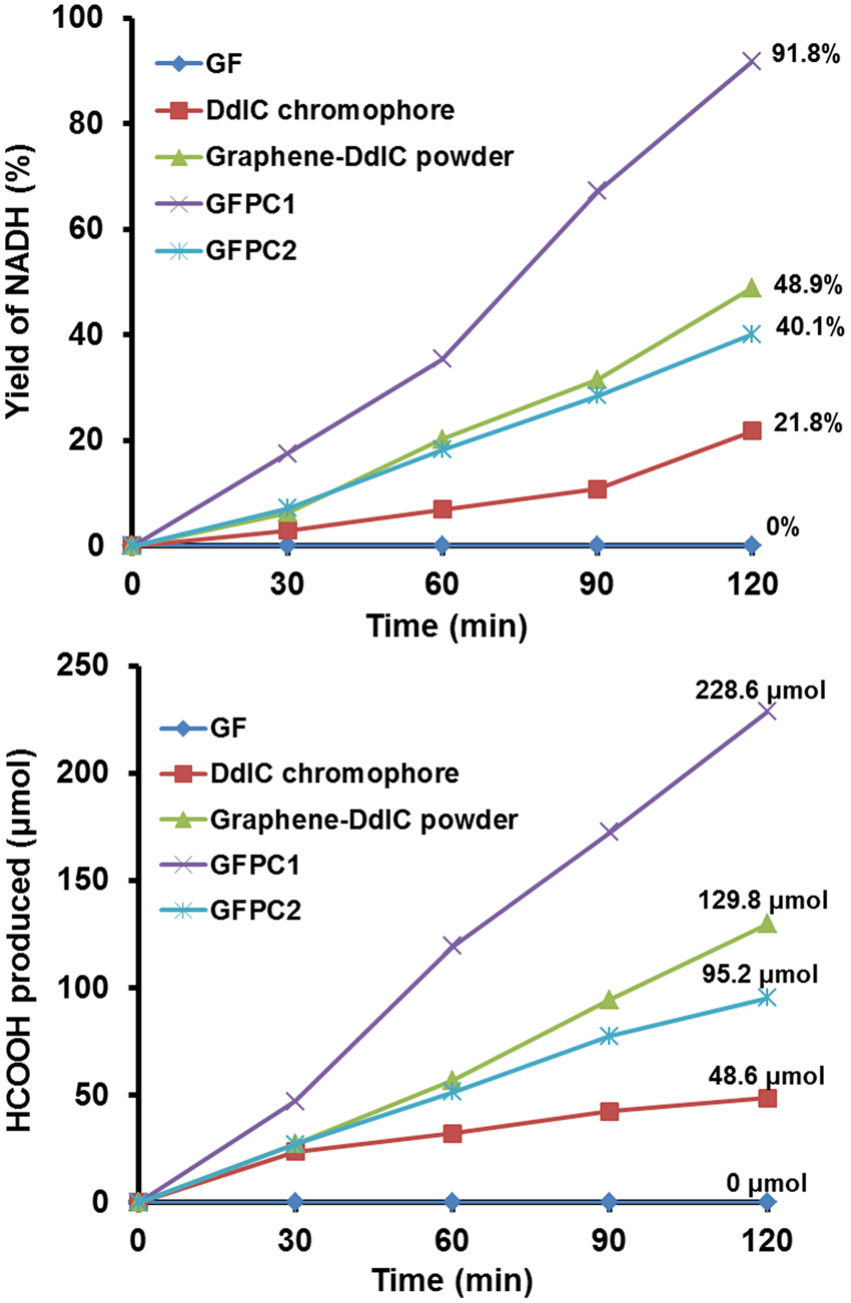
Photocatalytic activity of GF, DdIC chromophore, graphene-DdIC powder, GFPC 1 and GFPC 2. (**a**) NADH regeneration [β–NAD^+^ (1.24 μmol), **Rh** (0.62 μmol), AsA (0.1 mmol) and photocatalyst (1 × 1 cm^2^ film of GF, GFPC 1 and GFPC 2 or 0.5 mg of DdIC and graphene-DdIC powder) in 3.1 mL of sodium phosphate buffer (100 mM, pH 7.0)]. (**b**) Selective production of formic acid from CO_2_ (flow rate: 0.5 mL/min) under visible light [β–NAD^+^ (1.24 μmol), **Rh** (0.62 μmol), AsA (0.1 mmol), formate dehydrogenase (3 units) and photocatalyst (1 × 1 cm^2^ film of GF, GFPC 1 and GFPC 2 or 0.5 mg of DdIC and graphene-DdIC powder) in 3.1 mL of sodium phosphate buffer (100 mM, pH 7.0)].

The most critical part of the work was to evaluate the photocatalytic performance of the GFPC 1 in comparison to the DdIC chromophore, graphene-DdIC powder photocatalyst and its spin coated film sample (hereinafter GFPC 2; details in experimental section). Therefore, visible light-driven 1,4-NADH photo-regeneration ability of all these were examined along with GF. While GF failed to carry out any NADH regeneration, DdIC chromophore afforded 21.8% NADH regeneration over a period of 120 minutes. On the other hand, graphene-DdIC powder photocatalyst and GFPC 2 afforded 48.9 and 40.1% of NADH regeneration over a period of 120 minutes, respectively. Since GFPC 2 was obtained by spin coating the graphene-DdIC powder, a lower NADH regeneration by GFPC 2 can be clearly attributed to irregular orientation of the photocatalyst species on the polyimide sheet. In comparison to this 91.8% NADH regeneration was observed when GFPC 1 was evaluated for the photocatalytic activity (Fig. 5a). In other words, more than 225% higher NADH regeneration was carried out by GFPC 1 in the same time period when compared to spin coated GFPC 2.

To further examine the photocatalytic ability, they were used as the photocatalyst in an artificial photosynthetic system for formic acid production from CO_2_ (Fig. 2)^27^. The results obtained were similar to NADH regeneration. While as expected GF failed to carry out any formic acid formation, DdIC chromophore afforded 48.6 µmol of formic acid over a period of 120 minutes. On the other hand, graphene-DdIC powder photocatalyst and GFPC 2 afforded 129.8 and 95.2 µmol of formic acid over a period of 120 minutes, respectively. Similar to NADH regeneration results, GFPC 1 photocatalyst afforded 228.6 µmol of formic acid which is also 2.3 fold higher formic acid production compared to GFPC 2 in 120 minutes (Fig. 5b). Undoubtedly this remarkably higher photocatalytic activity of GFPC 1 can be attributed to the regular arrangement of light harvesting units with spatial orientation towards the visible light as well as improved delocalization of π electrons and direct electron transfer from covalently attached chromophore to monolayer graphene. At the same time it was also evident from these experiments that spin coated film sample show lower photocatalytic performance compared to powder type photocatalysts which can be attributed to irregular orientation of the photocatalyst species on the film substrate. Overall these experiments clearly verify the success of our new design and fabrication strategy for graphene film photocatalysts.

The stability and reusability of GFPC 1 photocatalyst were examined by subjecting it to 6 cycles of formic acid production from CO_2_. In comparison to the first cycle wherein the photocatalyst carried out 228.55 μmol of formic acid production, remarkably high 187.89 μmol of formic acid was obtained in the 6^th^ cycle (Fig. S7). These results indicate that GFPC 1 is a stable and reusable film photocatalyst for practical use.

The high formic acid production led us to consider the possibility of photocatalytic decomposition of GFPC 1 photocatalyst to carbon residues that may subsequently produce additional formic acid photocatalytically without CO_2_ consumption^28^. To discount this possibility, a control experiment in the absence of CO_2_ was performed. A lack of formic acid formation in this control experiment confirmed photocatalytic NADH regeneration under visible light irradiation. In a related fashion, a control experiment without the GFPC 1 photocatalyst (but with formate dehydrogenase enzyme) and another one without visible light irradiation were also carried out which also failed to show any detectable amount of formic acid. Additionally, no formic acid formation was detected in another set of control experiments carried out in the absence of rhodium complex **Rh** and/or NAD^+^ which confirmed their vital role in the functioning of the photocatalyst/biocatalyst integrated artificial photosynthesis system. To evaluate the possibility of rhodium complex **Rh** functioning as the CO_2_ reduction photocatalyst a control experiment consisting of rhodium complex and CO_2_ was performed. However, no formic acid was detected in this case also. This indicated that rhodium complex does not carry out photocatalytic CO_2_ reduction. Along with the high stability of GFPC 1 under visible light irradiation the various control experiments clearly point towards exclusive visible light driven photocatalytic NADH regeneration followed by enzymatic CO_2_ reduction as the sole route for formic acid production. This was further confirmed by C-13 isotope labelling experiment which clearly indicated that CO_2_ is the sole source of formic acid formation in our system (Fig. S8).

### Cyclic Voltammetry Studies

**Figure 6:**
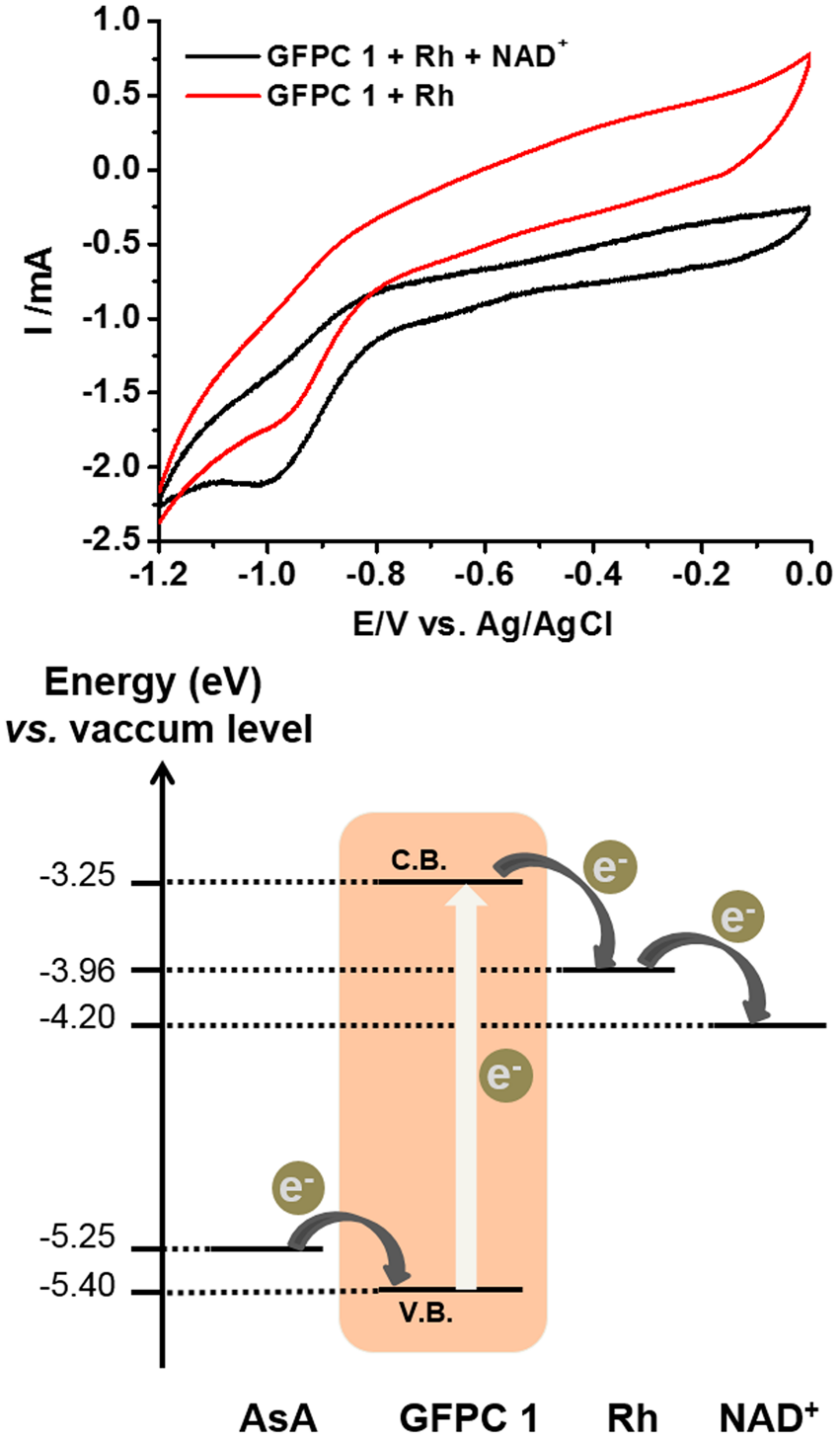
(**a**) Cyclic voltammetry (CV) studies on **Rh** and GFPC 1 in the absence and presence of NAD^+^. The potential was scanned at 100 mVs^−1^ using glassy carbon (working), silver-silver chloride (reference) and platinum (counter) electrodes in sodium phosphate buffer (100 mM, pH 7.0). (**b**) Proposed photocatalytic pathway based on CV studies^23,34,35^.

To understand the electron transfer pathway, cyclic voltammetry (CV) measurements were carried out. The reduction potential at cathodic peak current of GFPC 1 was observed at around −1.2 V (Fig. S9). On mixing GFPC 1 with rhodium complex **Rh** ([Cp*Rh(bpy)H_2_O]^2+^; Cp* = pentamethylcyclopentadienyl, bpy = 2,2′-bipyridine) reduction was observed at −0.97 V (Fig. 6a). This is an anodic shift of GFPC 1 reduction which is clearly indicative of electron transfer from GFPC 1 to **Rh**^23^. Moreover, the GFPC 1-**Rh** system revealed reduction potential at −1.05 V with NAD^+^, implying that the system consisting of GFPC 1 and **Rh** catalyzed the reduction of NAD^+^ to NADH (Fig. 6a). From these CV measurements it may be concluded that following the photoexcitation of the GFPC 1 electron from HOMO (E = −5.40 eV) to LUMO (E = −3.25 eV), it cascades into **Rh** (E = −3.79 eV). The vicinity and potential gradient between the light harvesting GFPC 1 photocatalyst and **Rh** center enable this efficient electron transfer from former to latter. The resultant reduced **Rh** species [Cp*Rh(bpy)] upon chemical protonation carries out catalytic regeneration of enzymatically active 1,4-NADH from NAD^+^ (E = −4.20 eV). This photocatalytic pathway is schematically shown in Fig. 6b.

### Photocurrent studies

**Figure 7:**
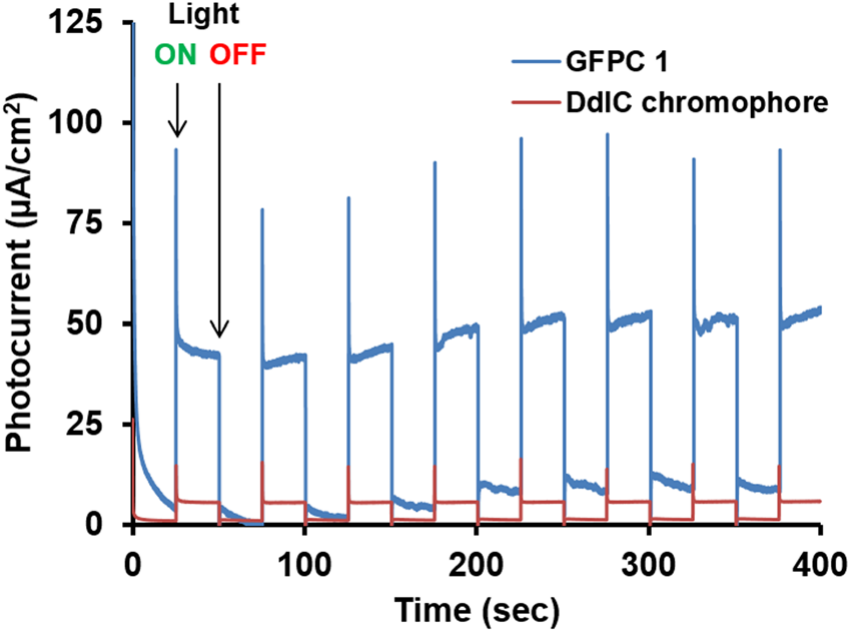
Photocurrent-time (I-T) profiles of FTO/GFPC 1 and FTO/DdIC electrodes under simulated solar light (1 sun) illumination (three electrodes; scan rate 50 mV/s; input power: 100 mW/cm^2^ and electrolyte: 0.1 M NaCl in water; bias potential: 0 to 0.1 V (vs Ag/AgCl)).

To further observe the behavior of the photogenerated electrons in GFPC 1, photocurrent measurements were carried out. The GFPC 1 film on FTO substrate exhibited a reversible photocurrent of 49 µA cm^−2^ in response to the on/off simulated sunlight (1 sun) illumination (Fig. 7). This value is nearly 10-times higher compared to that obtained for DdIC chromophore film on FTO substrate (5.1 µA cm^−2^) under identical conditions (Fig. 7). The high photocurrent is responsible for the photocatalytic activity of GFPC 1 under simulated solar light (one sun)^29^. Moreover photocurrent stability of GFPC 1 is evident from its reversible response on exposure to several on/off cycles. According to multiple reports this phenomenon indicates multi-electron transfer from DdIC to GF under one sun illumination^3,23,30,31^. It has also been experimentally demonstrated in a number of reports that graphene acts as an excellent multi electron transfer agent^3,23^. Thus, GFPC 1 can act as the photocatalytic conversion material for NADH regeneration and solar fuel formation^32^.

### Density Functional Theory calculations

**Figure 8:**
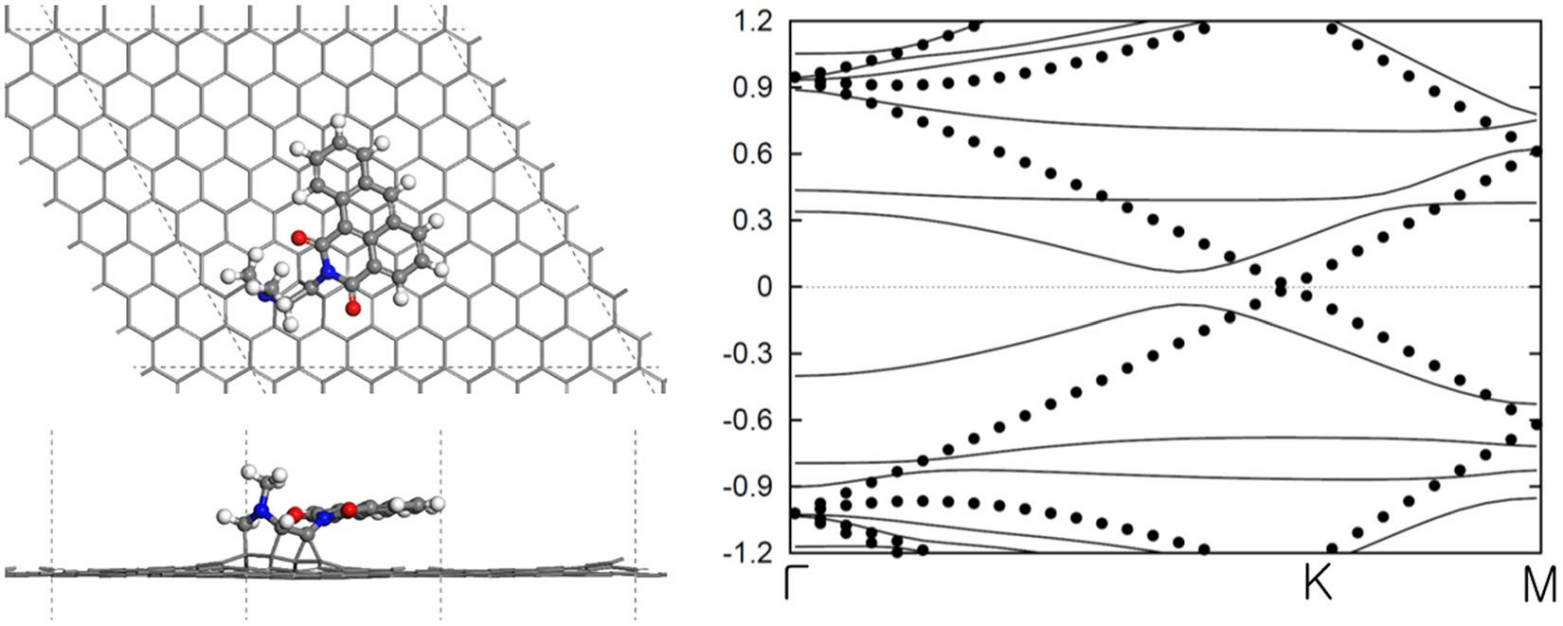
The optimized atomic geometry and the electronic band structure of GFPC 1. (**a**) Top-view and (**b**) side-view of GFPC 1. Note the π-π stacking between aromatic rings in DdIC chromophore and graphene. (**c**) The electronic band structure of GFPC 1 photocatalytic system. The electronic band of pristine graphene is also shown with circles. The hybridization between molecular orbitals in chromophore and graphene is noticeable around K point. The Dirac point of graphene lies around the center of HOMO-LUMO gap of the chromophore.

To elucidate the origin of the photocatalytic activity and activity enhancement of GFPC 1 theoretically, a series of first principles calculations using plane-wave based density functional theory (DFT) code, VASP were carried out^33^. At first, electronic structure calculations were performed separately on the DdIC chromophore and the graphene monolayer (an 8 × 8 unit composed of 128 carbon atoms). The Fermi level of graphene was calculated to be 4.20 eV lower than vacuum level, which lies around the center of bandgap of the chromophore, about 0.6 eV lower than the lowest unoccupied molecular orbital (LUMO) level of chromophore. Although this “divide-and-conquer” method is simple and powerful to deal with a large system without too much computational burden, the two components (DdIC chromophore and graphene monolayer) of the photocatalyst are tightly bonded *via* strong covalent bond. Thus the electron can be transferred spontaneously to make the Fermi levels equal. So, electronic structure calculations of the whole system were performed, secondly. The optimized atomic geometry and electronic band structure are shown in Fig. 8. The chromophore and graphene are tightly combined by four covalent bonds between two carbon atoms in chromophore and 4 atoms in graphene; the C-C bond lengths are within the range of 1.54–1.60 Å. The binding is further enhanced by π-π stacking between aromatic rings in chromophore and graphene. The band structure plot clearly shows that the Dirac point of graphene lies between the HOMO-LUMO energy-gap of the chromophore. The LUMO of chromophore lies ~0.3 eV higher than Dirac point. The localized molecular orbitals of chromophore are almost unaffected at Γ and M point but significantly hybridized with orbitals of graphene around K point. The incident light is absorbed at the chromophore, where occurs photoexcitation, and the created electrons move into the graphene via stable covalent bonds. The electron transfer efficiency could be estimated by the energy level alignment between the Fermi level of graphene and the LUMO of chromophore and also on the overlap of molecular orbitals. The existence of hybridized orbitals near Fermi level makes the ballistic transport of photoexcited electrons possible from the chromophore to graphene. The DFT calculation results clearly suggested that the photoexcited electrons transfer from chromophore finally to hydrogen reduction site via graphene. These simulation studies conform with the experimental results discussed above.

## Conclusion

In conclusion, the newly designed and fabricated flexible graphene film photocatalyst shows high photocatalytic activity attributed to a regular arrangement of light harvesting units with spatial orientation towards visible light as well as improved delocalization of π electrons and direct electron transfer from covalently attached chromophore to graphene monolayer. The graphene film based artificial photosynthesis system thus obtained shows remarkably higher formic acid production from CO_2_. We believe that this work would serve as a benchmark example of a new pool of graphene based photocatalysts that can efficiently harvest and transfer solar light and thereby trigger renewed interest in the area of solar fuel/chemical production from CO_2_. Our strategy may also provide broad contours for application of flexible graphene films in other fields such as solar energy powered wearable electronic devices.

## Materials and Methods

### Preparation of GFPC 1 photocatalyst

The GFPC 1 photocatalyst for this research work was obtained by coupling CVD grown graphene film with 1,3-Dioxo-1H-dibenzo[de,h]isoquinoline-2[3 H]-carbaldehyde (DdIC chromophore) via 1,3-dipolar cycloaddition. This was carried out by the following procedure.

Graphene film was suspended in 100 ml round-bottom flash via wire in 10 ml of ortho-dichlorobenzene (ODCB) along with sarcosine (2.5 mg), and DdIC (2.0 mg). The solution was stirred at 180 °C under argon atmosphere for 7 days. The GFPC 1 photocatalyst was then washed with ODCB, followed by water and CHCl_3_. The film was then dried in the oven at 125 °C for 24 hours to obtain GFPC 1 photocatalyst with the loading of one DdIC group per 70 carbon atoms of the graphene film.

### Preparation of graphene-DdIC powder photocatalyst and spin coated sample (GFPC 2) for comparison studies

Graphene for this sample was prepared by following a literature method^16^. The graphene thus obtained was coupled to DdIC chromophore by following the procedure as outlined for GFPC 1 except for the graphene powder used in this case. This sample is graphene-DdIC powder photocatalyst with the loading of one DdIC group per 68 carbon atoms of graphene.

A DMF suspension of the graphene-DdIC powder photocatalyst was spin coated (3000 rpm, 60 sec) on 1 × 1 cm^2^ polyimide sheet and then dried in an oven at 80 °C for 3 days to obtain GFPC 2.

### Photocatalytic NADH Production

The photochemical regeneration of NADH was performed within a quartz reactor under an inert atmosphere at room temperature, using a 450 W Xenon lamp (Newport 66921) with a 420 nm cut-off-filter as light source. The photocatalytic regeneration of NADH was carried out as follows. The reaction was performed in a quartz reactor. The reaction consisted of β–NAD^+^ (1.24 μmol), rhodium complex **Rh** (0.62 μmol), Ascorbic acid (0.1 mmol) and photocatalyst (1 × 1 cm^2^ film of GF, GFPC 1 and GFPC 2 or 0.5 mg of DdIC and graphene-DdIC powder) in 3.1 mL of sodium phosphate buffer (100 mM, pH 7.0). The regeneration of NADH was monitored by UV-vis spectrophotometer (UV-1800, Shimadzu).

### The artificial photosynthesis of formic acid from CO_2_

The artificial photosynthesis of formic acid from CO_2_ was also performed within a quartz reactor at room temperature, using a 450 W Xenon lamp with a 420 nm cut-off-filter as light source. The reaction consisted of photocatalyst (1 × 1 cm^2^ film of GF, GFPC 1 and GFPC 2 or 0.5 mg of DdIC and graphene-DdIC powder), β–NAD^+^ (1.24 µmol), rhodium complex **Rh** (0.62 μmol) and formate dehydrogenase enzyme (3 units) in 3.1 mL of sodium phosphate buffer (100 mM, pH 7.0) with Ascorbic acid (0.1 mmol) in the presence of CO_2_ (flow rate: 0.5 mL/min). The amount of formic acid was estimated by GC (7890 A, Agilent Technologies).

Note: The 1 × 1 cm^2^ film of GF, GFPC 1 and GFPC 2 contained 0.5 mg (approx.) of the photocatalyst.

### Density functional theory (DFT) calculations of electronic structures

All density functional theory (DFT) calculations were performed using the plane-wave approach as implemented in the VASP code within the generalized gradient approximation (GGA) using the Perdew-Burke-Ernzerhof (PBE) exchange-correlation function. Frozen-core projector augmented wave pseudopotentials were used. For the simulation of periodic system (8 × 8 unit cell of graphene), the Monkhorst-Pack scheme **k**-points sampling was done with 3 × 3 × 1 grid including Г-point for the integration of the irreducible Brillouin zone. For the molecular system, only Г-point is included. The Kohn–Sham wave functions of the valence electrons were expanded using a plane wave basis set within a specified energy cutoff that was chosen as 400 eV. The positions of the nuclei in the initial structures were first relaxed by the conjugate-gradient algorithm, until the Hellman-Feynman forces on each nucleus were lower than 0.01 eV/Å. After the relaxation, spin-polarized total energy of the electronic structure was calculated using a self-consistent field method that terminated when a change in total energy between two subsequent steps was less than 10^−6^ eV.

## Electronic supplementary material

Supplementary Information

## Data Availability

All pertinent data is presented within the manuscript. Raw data can be provided upon request as needed.
