# Supplementary material for: Highly Improved Solar Energy Harvesting for Fuel Production from CO2 by a Newly Designed Graphene Film Photocatalyst
Source: Sci Rep. 2018 Nov 13;8:16741. doi: 10.1038/s41598-018-35135-7 (PMC6233185; doi:10.1038/s41598-018-35135-7)
Supplement: Supplementary file 1 — Supplementary Information [file 41598_2018_35135_MOESM1_ESM.docx]

Electronic Supplementary Material

Highly Improved Solar Energy Harvesting for Fuel Production from CO_2_ by a Newly Designed Graphene Film Photocatalyst

Rajesh K. Yadav, Jeong-O Lee, Abhishek Kumar, No-Joong Park, Dolly Yadav, Jae Young Kim and Jin-Ook Baeg*

Artificial Photosynthesis Research Group, Korea Research Institute of Chemical Technology (KRICT), 100 Jang-dong, Yuseong, Daejeon 305 600, Republic of Korea

*E-mail: jobaeg@krict.re.kr

**1. General remarks**

Aceanthrylene-1,2-dione, Formamide, KOH, NaOH, HCl, Dichlorobenzene, H_2_SO_4_, H_2_O_2_, and CHCl_3_ were purchased from Aldrich. Cu foil (99.8%) was purchased from Alfa Aesar. Formate dehydrogenase enzyme and β–nicotinamide adenine dinucleotide were purchased from Sigma. All the solvents were of HPLC grade and used without further purification. Ultra-pure water was obtained using a Millipore System (Tech Sinhan Science). The organometallic mediator (**Rh**), [Cp*Rh(bpy)Cl]Cl, (Cp* = 5-C_5_Me_5_, bpy = 2,2-bipyridyl) was synthesized as described previously in literature.^1^

**2. Instruments and Measurements**

^1^H NMR spectra were recorded on a Bruker AVANCE II+ 300 MHz spectrometer with tetramethylsilane (TMS; δ = 0) as internal standard. The mass spectrum (EI-MS) was recorded on JEOL MStation JMS-700 mass spectrometer. UV-Visible spectra were recorded on Shimadzu UV-1800 spectrophotometer. Fourier transform infrared spectroscopy (FTIR) spectra were obtained on a Bruker ALPHA-T FT-IR spectrometer. The test specimens were prepared by the KBr-disk method. The thermogravimetric analysis was carried out on TA Instruments Q500 instrument over a range of 50-900 ºC with a heating rate of 10ºC min^-1^. XPS spectra were recorded on KRATOS Axis Nova photoelectron spectrometer. Raman spectra were recorded on Bruker SENTERA with a 50x objective lens and 532 nm laser excitation. A commercial atomic force microscope (Nanoscope; Digital Instruments, Veeco Metrology group), equipped with a J scanner was used to examine morphology of the sample in the tapping mode. SEM analysis was carried out on Tescan Mira 3 LMU FEG instrument (Accelerating voltage: 20kV). High-resolution transmission electron microscope (HRTEM) images were obtained on a FET Phillips instrument [Model No. 200k VLAB6, (FEL TECNAI G2-20S-Twin)] operated at 200 kV.

**3. Experimental Section**

**3.1. Synthesis of 1H,3H-Dibenzo[de,h]isochromene-1,3-dione (Anthracene-1,9-dicarboxylic acid anhydride)**

Anthracene-1,9-dicarboxylic acid anhydride was prepared by a reported method.^2^ Aceanthrylene-1,2-dione (1.115 mmol), 1,4-dioxane (25 mL), and 2N NaOH (7 mL) were mixed with 30% hydrogen peroxide (5.6 mL), water (25 mL), and 2N H_2_SO_4_ (48.2 mL), left over night with stirring and then allowed to stand for one day. The light yellow precipitate transformed into orange colour, which was collected by filtration. The filtered compound was dissolved in 2N KOH solution, and acidified with 37% HCl leading to a yellow precipitate. The product thus obtained was filtrated, washed several times with distilled water (250 ml), and left to dry at 120ºC for one day in an oven to give an orange colour powder as the final product (1.03g, 79%). The characterization data for the synthesized compound corresponded well with the literature data.^2^

**3.2. Synthesis of 1,3-Dioxo-1H-dibenzo[de, h]isoquinoline-2[3H]-carbaldehyde (DdIC):**

Anthracene-1,9-dicarboxylic acid anhydride (3.88 g, 15.64 mmol) in formamide solution (150 mL) was refluxed at 210ºC for 5 h, further treated with formamide solution (50 mL), and heated at reflux for 14 h, followed by careful acidification with 37% HCl. The product thus obtained was filtered (sintered glass filter), thoroughly washed with distilled water (250 mL) and then dried in oven at 120ºC for one day. The product was then purified by column chromatography (silica gel, chloroform) to afford a bright yellow powder as final product (2.1g, 58.46%).^2^  ^1^H NMR (300 MHz, DMSO-d_6_, δ values in ppm): d=7.80 (m, 1H_aromatic H_), 7.93 (m, 1H_aromatic H_), 8.01 (m, 1H_aromatic H_), 8.40 (d, J=6 Hz, 1H_aromatic H_), 8.70 (d, J=5.5 Hz, 1H_aromatic H_), 8.72 (d, J=6.0 Hz, 1H_aromatic H_), 9.37 (s,1H_aromatic H_), 9.58 (d, J=10.0 Hz, 1H_aromatic H_); EI-MS (m/z): 275.15.

**3.3. Growth and transfer of graphene film**

Monolayer graphene was grown on a Cu foil (cut into 6×6 cm^2^ to fit in a 4 inch tube furnace) using chemical vapour deposition (CVD). Prior to CVD, Cu foils were cleaned with Ni etchant for 5-10 min, and rinsed with DI water. Cleaned Cu foil was loaded in the 4 inch hot-wall furnace, evacuated, heated to 1000°C and annealed for 20 min under 100 sccm H_2_ flow, in order to remove any contaminants or oxidized Cu. Graphene growth was initiated by introducing 30 sccm CH_4_ and 30 sccm H_2_ into the chamber, and two step growth process consists of 40 min exposure to 30 sccm CH_4_ and 30 sccm H_2_ followed by 40 min exposure to CH_4_ was performed. Growth process was terminated by evacuating the chamber and cooling to RT. CVD-grown monolayer graphene was transferred to target polyimide film with the aid of poly(methyl methacrylate) (950K, 4% volume dissolved in chlorobenzene) solution. Briefly, PMMA solution was spin coated on the graphene grown on a Cu foil, and then dried at room temperature for about 30 min. Cu foil was etched using the Cu etchant solution, the resulting PMMA-graphene film was collected, and cleaned several times by re-suspending the film in a clean DI water bath. Finally, suspended PMMA-graphene film was drawn on to the target polyimide substrate and dried overnight at ambient conditions. Erasing PMMA on graphene using acetone solution provided flexible graphene film ready for further functionalization.^3^

**3.4. Preparation of graphene film coupled to DdIC chromophore photocatalyst (GFPC 1)**

The GFPC 1 photocatalyst for this research work was obtained by coupling CVD grown graphene film with 1,3-Dioxo-1H-dibenzo[de,h]isoquinoline-2[3H]-carbaldehyde (DdIC chromophore) via 1,3-dipolar cycloaddition.^4^ This was carried out by the following procedure.

Graphene film was suspended in 100 ml round-bottom flash via wire in 10 ml of ODCB along with sarcosine (2.5 mg), and DdIC (2.0 mg). The solution was stirred at 180ºC under argon atmosphere for 7 days. The GFPC 1 photocatalyst was then washed with ODCB, followed by water and CHCl_3_. The film was then dried in the oven at 125ºC for 24 hours.

**3.5. Preparation of graphene-DdIC powder photocatalyst and spin coated sample (GFPC 2) for comparison studies**

Graphene for this sample was prepared by following a literature method.^5^ The graphene thus obtained was coupled to DdIC chromophore by following the procedure as outlined for GFPC 1 except for the graphene powder used in this case. This sample is graphene-DdIC powder photocatalyst.

A DMF suspension of the graphene-DdIC powder photocatalyst was spincoated (3000 rpm, 60 sec) on 1x1 cm^2^ polyimide sheet and then dried in oven at 80ºC for 3 days to obtain GFPC 2.

**4.1. Photoelectrochemical measurements:** Photoelectrochemical measurements were carried out in a three-armed cell consisting of reference (Ag/AgCl, BASI, MF-2063 RE-5), working (GFPC 1 electrode) and Platinum wire (Part Number: CHI115) counter electrodes using a Electrochemical analyzer (CHI Instruments 1100A). 0.1 M NaCl solution containing 0.1 M ascorbic acid was used as a redox couple/electrolyte. Newport solar simulator (69911) was used as a light source. Light intensity was measured by VSLI standard incorporated Oriel P/N 91150V. For CV studies- Potential scanned at 100 mVs^-1^ scan rate.

For photocurrent studies- 50 mVs^-1^ scan rate; bias potential: 0 to 0.1 V (vs. Ag/AgCl).

The GFPC 1 electrode was prepared by transferring CVD-grown monolayer graphene onto FTO glass. This was then coupled to the chromophore by following a similar procedure as outlined above for GFPC 1.

**4.2. Photocatalytic NADH Production:** The photochemical regeneration of NADH was performed within a quartz reactor under an inert atmosphere at room temperature, using a 450W Xenon lamp (Newport 66921) with a 420 nm cut-off-filter as light source. The photocatalytic regeneration of NADH was carried out as follows. The reaction was performed in a quartz reactor. The reaction consisted of β–NAD^+^ (1.24 μmol), rhodium complex **Rh** (0.62 μmol), Ascorbic acid (0.1 mmol) and photocatalyst (1x1 cm^2^ film of GF, GFPC 1 and GFPC 2 or 0.5 mg of DdIC and graphene-DdIC powder) in 3.1 mL of sodium phosphate buffer (100 mM, pH 7.0). The regeneration of NADH was monitored by UV-vis spectrophotometer (UV-1800, Shimadzu).

**4.3. The artificial photosynthesis of formic acid from CO_2_:** The artificial photosynthesis of formic acid from CO_2_ was also performed within a quartz reactor at room temperature, using a 450W Xenon lamp with a 420 nm cut-off-filter as light source. The reaction consisted of photocatalyst (1x1 cm^2^ film of GF, GFPC 1 and GFPC 2 or 0.5 mg of DdIC and graphene-DdIC powder), β–NAD^+^ (1.24 µmol), rhodium complex **Rh** (0.62 μmol) and formate dehydrogenase enzyme (3 units) in 3.1 mL of sodium phosphate buffer (100 mM, pH 7.0) with Ascorbic acid (0.1 mmol) in the presence of CO_2_ (flow rate: 0.5 mL/min). The amount of formic acid was estimated by GC (7890A, Agilent Technologies).

Note: The 1x1 cm^2^ film of GF, GFPC 1 and GFPC 2 contained 0.5 mg (approx.) of photocatalyst.

**4.4. Quantum Efficiency determination:** The Quantum efficiency determination of GFPC 1 for photocatalytic NADH regeneration was carried out in a quartz cuvette reactor (3.5 mL) at room temperature. A 450-watt Newport Xenon Lamp (Oriel) attached to an IR filter (Oriel), and a 20 nm bandpass filter at 420 nm was used as a light source. The number of incident photons was measured using a silicon photodiode with integrating sphere (Oriel).

The photochemical regeneration of NADH was conducted by illuminating the quartz reactor containing β–NAD^+^ (1.24 µmol), rhodium complex **Rh** (0.62 µmol), Ascorbic acid (0.1 mmol) and 1x1 cm^2^ film of GFPC 1 in 3.1 ml of 0.1 M sodium phosphate (NaH_2_PO_4_-Na_2_HPO_4_) buffer (pH ~ 7.0). The concentration of NADH was spectrophotometrically measured through the change in absorbance of NADH at 340 nm in UV-visible spectrum. The quantum efficiency was calculated using the equation below:

The quantum efficiency was calculated to be 17.4 % for the photocatalytic NADH regeneration process.

**5. Density functional theory calculations of electronic structures**

All density functional theory (DFT) calculations was performed using the plane-wave approach as implemented in the VASP code^6^ within the generalized gradient approximation (GGA) using the Perdew-Burke-Ernzerhof (PBE) exchange-correlation function.^7^ Frozen-core projector augmented wave pseudopotentials^8,9^ were used. For the simulation of periodic system (8x8 unit cell of graphene), the Monkhorst-Pack scheme **k**-points sampling was done with 3x3x1 grid including Г-point for the integration of the irreducible Brillouin zone. For the molecular system, only Г-point is included. The Kohn–Sham wave functions of the valence electrons were expanded using a plane wave basis set within a specified energy cutoff that was chosen as 400 eV. The positions of the nuclei in the initial structures were first relaxed by the conjugate-gradient algorithm, until the Hellman-Feynman forces on each nucleus were less than 0.01 eV/Å. After the relaxation a spin-polarized total energy of the electronic structure was calculated using a self-consistent field method that terminated when change in total energy between two subsequent steps was less than 10^−6^ eV.

For the accurate estimation of work function, we have adopted the dipole correction, proposed by Neugabeauer and Scheffler.^10^ The correction removes the artificial field arising in the empty space from the use of Periodic Boundary Conditions (PBC) in solution of Poisson equation by Fast Fourier Transform (FFT) method.

**Figures**

**(a)**

**
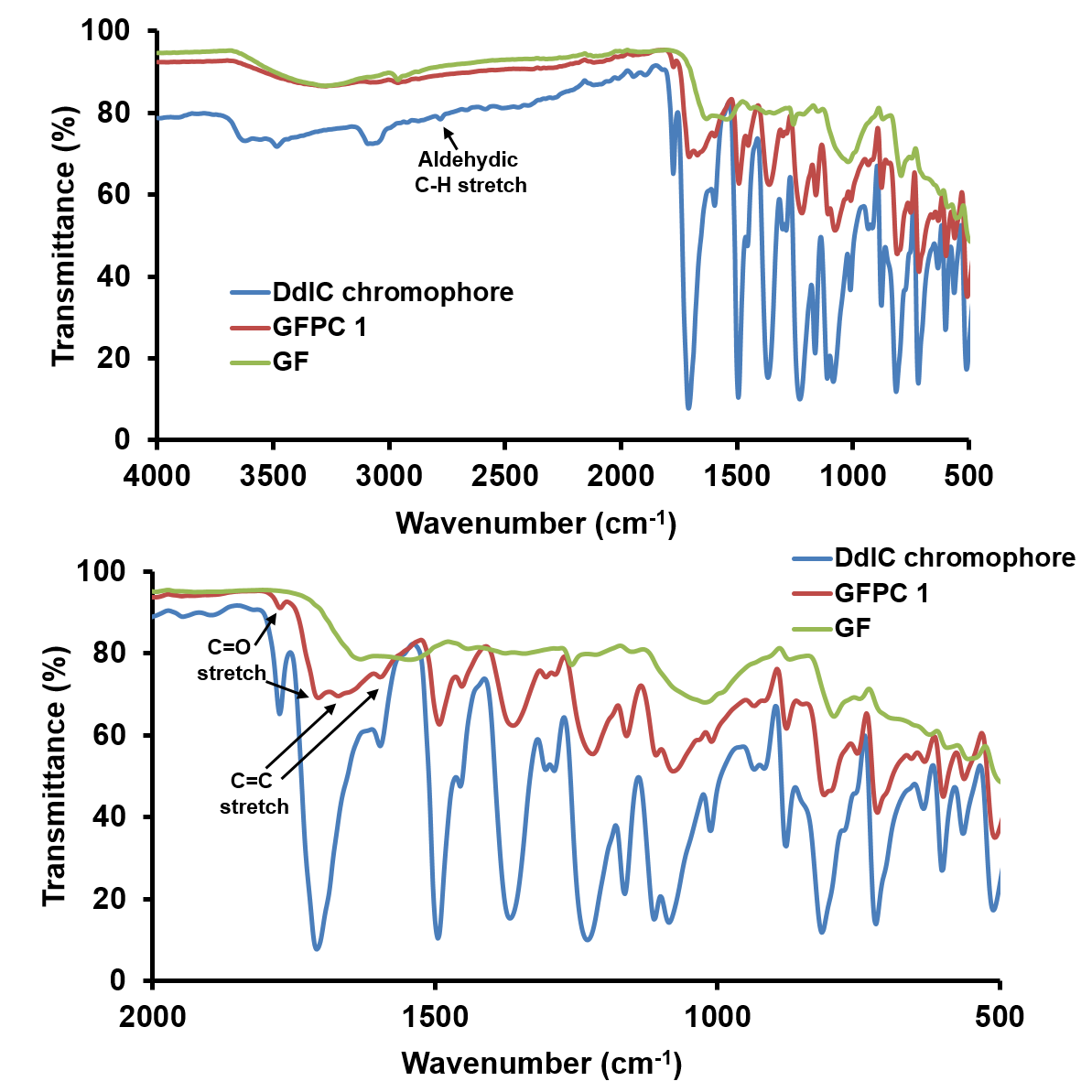
**

**(b)**

**Figure S1.** (a) FTIR spectra of DdIC chromophore, GFPC 1 and GF. (b) Expanded portion (2000-500 cm^-1^) of FTIR spectrum of DdIC chromophore, GFPC 1 and GF.

**
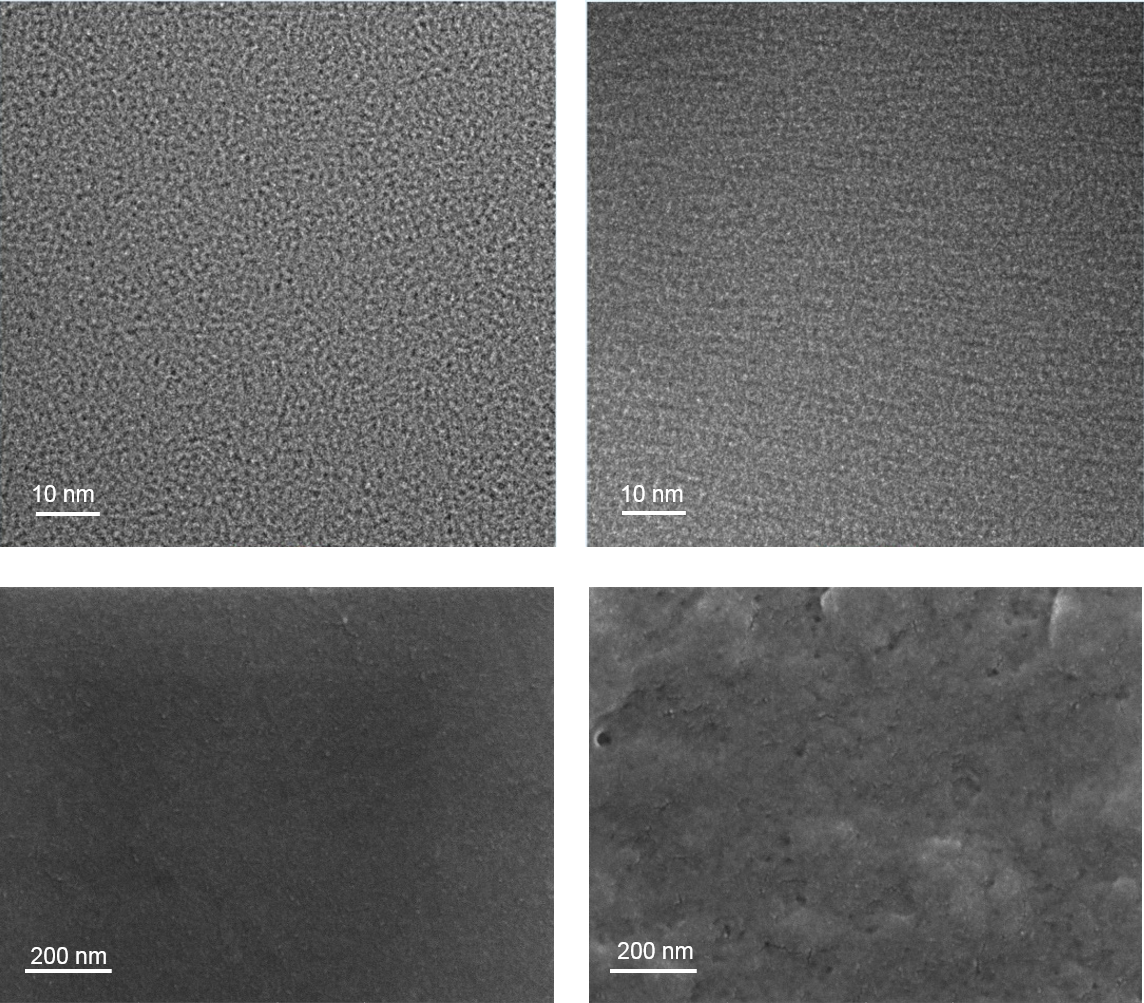
**

**(d)**

**(c)**

**(b)**

**(a)**

**Figure S2.** (a) HRTEM image of GF. (b) HRTEM image of GFPC 1. (c) SEM image of GF. (d) SEM image of GFPC 1.

**
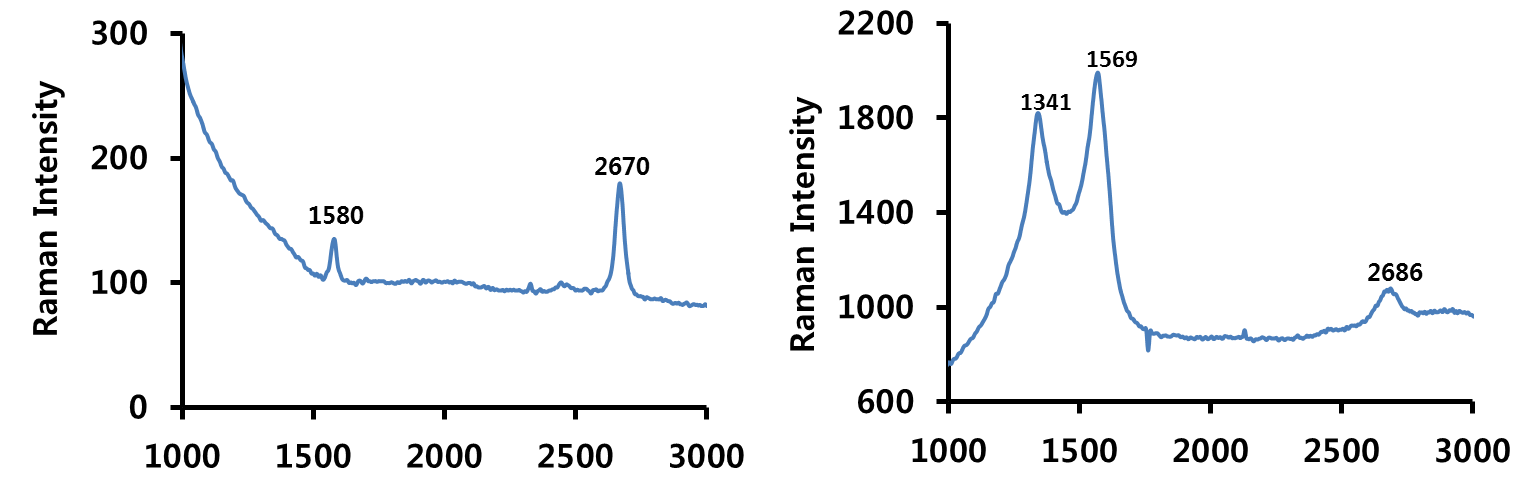
**

**(b)**

**(a)**

**Figure S3.** Raman spectra of (a) GF and (b) GFPC 1.

**Figure S4.** N1s X-ray photoelectron spectra (XPS) of GFPC 1.

**(a)**

**(b)**

**Figure S5.** C1s X-ray photoelectron spectra (XPS) of (a) GF and (b) GFPC 1.

**
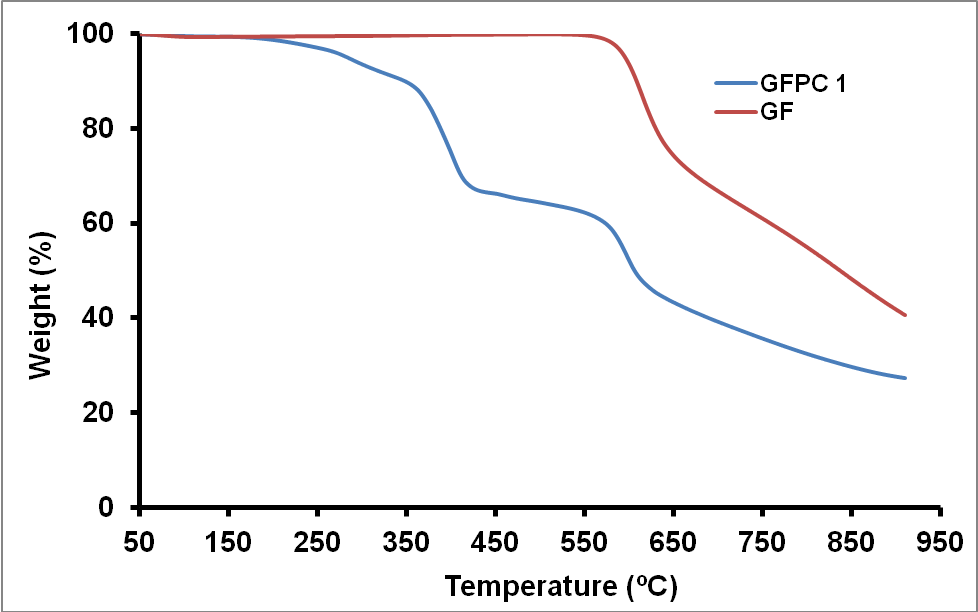
**

**Figure S6.** TGA thermograms of GF and GFPC 1.

**
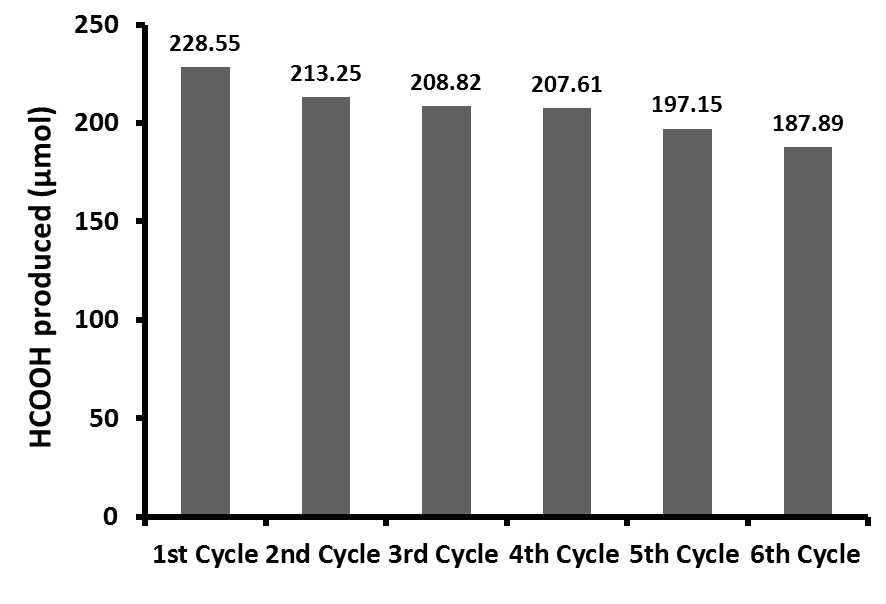
**

**Figure S7.** Formic acid production from CO_2_ upon repetitive use (6 cycles) of GFPC 1 photocatalyst [β–NAD^+^ (1.24 μmol), **Rh** (0.62 μmol), AsA (0.1 mmol) and 1x1 cm^2^ film of GFPC 1 photocatalyst in 3.1 mL of sodium phosphate buffer (100 mM, pH 7.0)].


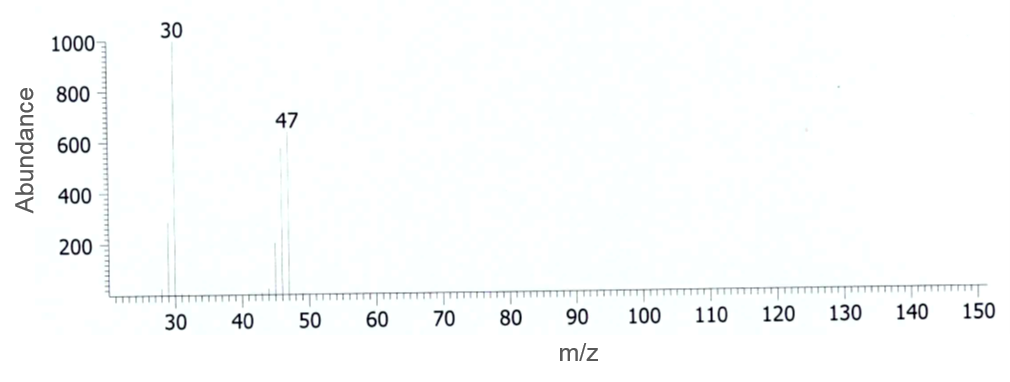


**Figure S8.** GC-MS spectrum of formic acid (H^13^COOH) obtained from ^13^CO_2_ using GFPC 1 photocatalyst-biocatalyst integrated artificial photosynthetic system.

**Figure S9.** Cyclic voltammogram (CV) of GFPC 1.

**References**

1. Lee, S. H., Nam, D. H., Kim, J. H., Baeg, J.-O. & Park, C. B. Eosin Y‐Sensitized Artificial Photosynthesis by Highly Efficient Visible‐Light‐Driven Regeneration of Nicotinamide Cofactor. *ChemBioChem* **10**, 1621 – 1624 (2009).

## 2. Langhals, H., Schönmann, G. & Polborn, K. Anthracene Carboxyimides and Their Dimers. *Chem. Eur. J.* **14**, 5290-5303 (2008).

3. Choi, W. J., Chung, Y. J., Park, S., Yang, C.-S., Lee, Y. K., An, K.-S., Lee, Y.-S. & Lee, J.-O. A Simple Method for Cleaning Graphene Surfaces with an Electrostatic Force. *Adv. Mater.* **26**, 637-644 (2014).

4. Zhang, X., Hou, L., Cnossen, A., Coleman, A. C., Ivashenko, O., Rudolf, P., van Wees, B. J., Browne, W. R. & Feringa, B. L. One‐Pot Functionalization of Graphene with Porphyrin through Cycloaddition Reactions. *Chem. Eur. J.* **17**, 8957-8964 (2011).

# 5. Gao, W., Alemany, L. B., Ci, L. & Ajayan, P. M. New insights into the structure and reduction of graphite oxide. *Nature Chem.* **1**, 403 – 408 (2009).

6. Kresse, G. & Furthmuller, J. Efficient iterative schemes for *ab initio* total-energy calculations using a plane-wave basis set. *Phys. Rev. B.* **54**, 11169-11186 (1996).

### 7. Perdew, J. P., Burke, K. & Ernzerhof, M. General Gradient Approximation Made Simple. *Phys. Rev. Lett.* 77, 3865-3868 (1996).

### 8. Blochl, P. E. Projector Augemented-Wave Method. *Phys. Rev. B.* 50, 17953-17979 (1994).

### 9. Kresse, G. & Joubert, D. From ultrasoft pseudopotentials to the projector augmented-wave method. *Phys. Rev. B.* 59, 1758-1775 (1999).

### 10. Neugebauer, J. & Scheffler, M. Adsorbate-substrate and adsorbate-adsorbate interactions of Na and K adlayers on Al(111). *Phys. Rev. B.* 46, 16067 (1992).
